# Children’s exposure to unhealthy food and beverage advertising in Senegal: a cross-sectional mixed-methods analysis of advertising trends, marketing strategies and perceptions

**DOI:** 10.1080/16549716.2026.2723719

**Published:** 2026-09-04

**Authors:** Yassarath Onifade, Adama Diouf, Olouwafemi Mistourath Mama, Stefanie Vandevijvere, Abdou Badiane, Ndeye Ndambao Sarr, Sylvain Landry Birane Faye, Julien Soliba Manga, Jean-Claude Moubarac, Nicole Idohou-Dossou

**Affiliations:** a Laboratoire de Recherche en Nutrition et Alimentation Humaine (LARNAH), Faculté des Sciences et Techniques (FST), Université Cheikh Anta Diop (UCAD), Dakar, Senegal; b Department of Epidemiology and Public Health, Scientific Institute of Public Health, Sciensano, Brussels, Belgium; c Laboratoire de Sociologie, d’Anthropologie et de Psychologie (LASAP-ETHOS), Faculté des Lettres et Sciences Humaines (FLSH), Université Cheikh Anta Diop (UCAD), Dakar, Senegal; d Département de Nutrition, TRANSNUT et CRESP (Centre de Recherche en Santé Public), Université de Montreal, Montreal, Canada

**Keywords:** School environments, nutrient profiling, obesogenic environments, television food advertising, public health policy, food marketing regulation

## Abstract

**Background:**

Marketing of nutrient-poor foods is a recognized driver of unhealthy dietary behaviors and childhood obesity. In Senegal, the triple burden of malnutrition persists, while regulations restricting unhealthy food marketing to children remain limited.

**Objective:**

To assess the extent and nature of food advertising around schools and on television in Senegal and to explore children’s and parents’ perceptions of food marketing.

**Methods:**

A cross-sectional mixed-methods study was conducted across four regions in Senegal (Dakar, Kaolack, Saint-Louis, Ziguinchor). Food advertising was mapped within a 250-m radius of 40 selected urban/peri-urban schools, and inventoried on four national television channels. Advertised foods were evaluated using the WHO-NPM AFRO and Nutri-Score systems and classified as ‘permitted’ or ‘non-permitted’ foods. Focus group discussions with children and in-depth interviews with parents were conducted to explore food marketing perceptions. Quantitative data were analyzed using descriptive and inferential statistical analysis, and qualitative data were analyzed using thematic analysis.

**Results:**

Overall, 79% (*n* = 438) of food advertisements around schools and 75% (*n* = 366) on television were for ‘non-permitted’ foods. Non-permitted food advertisements were more frequent during peak viewing times and used persuasive marketing techniques. Children demonstrated brand recall and reported requesting advertised products following exposure. Parents expressed concerns about poor nutritional quality of foods advertised to children and supported stricter regulatory measures.

**Conclusions:**

Children were exposed to ‘non-permitted’ food advertising across physical and virtual environments in Senegal. The predominance of nutrient-poor food promotion underscores the urgent need for mandatory policies to restrict unhealthy food marketing to children.

## Paper Context

**Main findings**: Children are highly exposed to food marketing through multiple settings, particularly around schools and on television. Most advertised products are nutrient-poor foods, and participants perceived marketing as influencing food preferences and consumption behaviors.

**Added knowledge**: This study provides a comprehensive assessment of children’s exposure to food marketing in Senegal by combining measures of advertising exposure with subjective experiences of how food marketing shapes children’s food environments.

**Global health impact for policy and action**: Findings support comprehensive restrictions on unhealthy food marketing to children and provide evidence to strengthen food environment policies in Senegal to prevent childhood obesity and promote healthier food environments in line with WHO recommendations.

## Background

Senegal is experiencing a nutritional transition and faces a persistent triple burden of malnutrition. Among children under 5 years, 18% are stunted, 8% are wasted and 14% are underweight [1]. Micronutrient deficiencies remain highly prevalent, with nearly 60% of children aged 6–59 months affected by anemia. In parallel, a shift toward overnutrition is evident, particularly in urban settings, where 3% of children under 5 years are overweight [1,2]. A similar pattern is observed among adolescents aged 10–19 years, with persistent underweight (21.7%) alongside rising overweight (5.9%) and abdominal obesity (5.0%) [3].

One of the major drivers of this evolving nutritional landscape is the rapid transformation of food environments. Food environments encompass the collective physical, economic, political, and socio-cultural conditions shaping dietary behaviors and nutritional status [4]. Across many African countries, these changes have accelerated the nutrition transition, characterised by a shift towards diets increasingly dominated by energy-dense, nutrient-poor foods, particularly ultra-processed foods (UPFs) [5,6]. UPFs are industrial formulations made predominantly from food-derived substances, with little or no whole foods, and typically containing a range of additives [7]. Evidence from Senegal indicates the increasing availability of UPFs within the food system [8,9], which is of concern because higher consumption of these products has consistently been associated with poorer diet quality, energy, nutrient intakes, and therefore an increased risk of obesity and other adverse health outcomes [10–13].

Food marketing is a key driver of obesogenic food environments, shaping children’s food preferences, purchase requests and consumption patterns [14–17]. Children are particularly vulnerable due to their limited ability to recognize persuasive intent [18]. Evidence from multiple African contexts consistently shows that food advertising predominantly promotes unhealthy foods, particularly products high in fat, sugar and salt (HFSS), and UPFs [19–22]. Exposure is often higher in socio-economically disadvantaged neighborhoods [20], reflecting broader inequalities in local food environments. Repeated exposure to such marketing has been associated with increased consumption of unhealthy foods, poorer diet quality, stronger brand recognition [15,16], and a higher risk of overweight and obesity among children and adolescents [23]. A recent UNICEF analysis of digital food marketing in Eastern and Southern Africa found that approximately 96–99% of advertised products did not meet WHO nutritional standards [6], highlighting the dominance of nutrient-poor foods in children’s food environments. A similar pattern has been reported in Kenya, where nearly 88% of products marketed to children on Instagram did not meet the criteria for advertising for children. Of these, 60% appealed to younger children and 66% to adolescents [24].

Despite the well-established health impacts of exposure to marketing of HFSS foods among children, policy responses remain limited. Restricting the marketing of unhealthy foods to children is recognized as a cost-effective ‘Best Buy’ intervention for NCDs prevention [12]. Comprehensive restrictions across physical and digital environments, supported by nutrient profiling and effective enforcement mechanisms, have been recommended [6,25]. However, implementation remains weak in many low- and middle-income countries (LMICs) [26].

In Senegal, the regulatory framework governing advertising is primarily based on general provisions, including Law No. 83–20 on advertising, which establishes general requirements for advertising practices but does not provide specific restrictions on the marketing of unhealthy foods and beverages to children. While Senegal’s national nutrition strategies recognize the importance of early-life nutrition [27], they have primarily focused on programme-based interventions, such as promoting breastfeeding, improving complementary feeding practices and addressing micronutrient deficiencies, rather than enforceable regulatory measures to protect children from unhealthy food marketing. Beyond these programme-based approaches, some progress has recently been made with the adoption of Decree No. 2025–751, which regulates the marketing of breast-milk substitutes and complementary foods for infants and young children [28]. This regulatory gap highlights the need for context-specific evidence to inform the development of effective policies to protect children from unhealthy food marketing.

The International Network for Food and Obesity/Non-Communicable Disease Research, Monitoring and Action Support (INFORMAS) has developed standardized methods to assess food environments, including modules for monitoring outdoor and television advertising [29]. Although evidence from Africa remains limited, emerging studies have documented children’s exposure to unhealthy food marketing [19–22]. National evidence generated using standardized methods is essential to inform policy and strengthen regulatory action.

Recognising the harmful impact of unhealthy food marketing on children’s health, this study aimed to: (i) describe the food advertising landscape around schools and on the most-watched television channels in Senegal; (ii) assess the nutritional quality of advertised foods using the WHO Nutrient Profile Model for the African Region (WHO-NPM AFRO) and the Nutri-Score classification system; and (iii) explore children’s and parents’ perceptions and experiences of food marketing.

## Methods

### Study design

We conducted a cross-sectional mixed-methods study using the INFORMAS framework [29]. The study comprised three components: an environmental audit of outdoor food and beverage advertising around schools, a television advertising inventory, and a qualitative component exploring the perceptions and experiences of food marketing among children, adolescents, parents or home-caregivers, teachers, school directors and food vendors. This mixed-methods approach provided a comprehensive assessment of the food marketing environment for children by integrating quantitative evidence on the extent and nature of food advertising with qualitative insights into the perceptions and experiences of children, parents, and other key stakeholders in the school food environment.

### Study setting and sampling

#### Outdoor advertising around schools

Between May and June 2022, we conducted a cross-sectional mapping of food and beverage advertisements around primary and secondary schools in four regions of Senegal: Dakar (West), Kaolack (Centre), Saint-Louis (North), and Ziguinchor (South). These regions were purposively selected to capture geographical and urbanization diversity. Dakar is the country’s largest metropolitan area, while the other regions include secondary cities and mixed urban and peri-urban settings.

A list of all registered schools (elementary, middle and secondary) in the four target regions was obtained from the Ministry of Education, which served as the sampling frame. Within each region, schools were randomly selected using a stratified approach to ensure balanced representation across school level (elementary/secondary), status (public/private), and area (urban/peri-urban). In total, 40 schools were selected. In Kaolack, Saint-Louis and Ziguinchor, eight schools per region were included (two elementary and two secondary schools in both urban and peri-urban areas). Due to the concentration of the urban population and commercial activity in Dakar, 16 schools (eight elementary and eight secondary) were randomly selected from two departments, which were purposively selected because of their high population density and to capture different socio-economic contexts within the country’s largest metropolitan area. The number of schools selected was determined by the requirement to achieve sufficient variation within these strata, while ensuring that detailed in-person geographic mapping remained feasible.

A 250-m Euclidean buffer was generated around the main entrance of each school using Arc-GIS software, in order to define the school food marketing environment. This radius was selected based on prior research examining school food environments and reflects a distance that can easily be walked by students before and after school. All food and beverage advertisements within each school buffer were systematically recorded using the INFORMAS food promotion module for monitoring outdoor food advertising [28].

#### Television advertising monitoring

Between May and July 2023, an inventory of food and beverage advertising was conducted on the four television channels in Senegal with the highest audience reach (one public and three private), as selected based on Mediametrie audience data [30], which provides information on awareness, and audience viewership for television channels in Senegal.

The selected channels’ television broadcasts were continuously recorded from 6.00am to midnight over eight non-consecutive days, excluding national holidays. This timeframe was chosen to capture peak viewing times, which are defined as the top five 1-h time slots with the highest average audience viewership on weekdays and weekends [29]. In Senegal, these periods correspond to 13:00–15:00 and 19:00–22:00, based on national audience data [30].

The recording schedule included four weekdays (Monday, Tuesday, Thursday and Friday) and four weekend days (two Saturday and two Sunday), to allow for variation in advertising patterns throughout the week. Each channel was recorded for 8 days, with one recording per day, to minimize duplication of programming and ensure diversity of broadcast content. The recordings followed a structured rotational schedule, ensuring that each channel was captured on eight different days, including weekdays and weekends (see Supplementary Figure S1). The recordings were conducted using a digital video recorder and stored on an external hard disk for subsequent coding and analysis.

#### Qualitative component

Focus group discussions (FGDs) and in-depth interviews (IDIs) were conducted in the four regions. Participants were recruited, with the support of school directors, from a sub-sample of the previously selected schools.

A total of 209 schoolchildren and adolescents participated in 24 FGDs (~8–10 participants per group), which were stratified by age and sex to facilitate open discussion. In elementary schools, students in 2^nd^ to 5th grade (~8–11 years) were recruited, as children in this age range were considered capable of meaningfully engaging with the discussion topics. Additionally, 26 IDIs were conducted with parents or home-caregivers (*n* = 7), teachers and school directors (*n* = 14), and food vendors (*n* = 5) operating within or around the selected schools, in order to capture a range of perspectives. Recruitment continued until sufficient diversity of perspectives was achieved. The distribution of FGDs and qualitative participants across regions is presented in Supplementary Table S1.

All FGDs and IDIs were facilitated by trained socio-anthropologists using a semi-structured interview guide informed by previous qualitative research on food marketing [31–33]. The discussions explored exposure to food advertising, perceptions of marketing techniques and the perceived effects on food preferences and purchasing behaviors. Sessions were conducted in French or local language (Wolof), according to participants’ preference, audio-recorded, and transcribed verbatim. Field notes were completed after each session to document contextual observations and reflexive considerations.

#### Data collection procedures

##### Food advertisements around schools

Three trained research assistants photographed and geolocated food and beverage advertisements located within the 250-m buffer, using a structured observational tool. The observational tool captured data collected on the location, size and format of the advertisement, product(s) promoted, brand/company name, the categorization of the product based on the WHO-NPM AFRO food categories (Table S2). Additional categories were created for products not explicitly covered by the model, including infant formula. Advertisements displaying only company or brand logo, with no specific product, were coded as ‘brand-only’ advertisements. Following data collection, advertisement images were reviewed to verify and complete field coding.

Two researchers conducted detailed post hoc coding of persuasive marketing techniques (e.g. the use of promotional characters, premium offers, brand benefit claims, health and/or nutrition-related claims, and emotional appeals) and jointly reviewed all advertisements to ensure consistency. These techniques were coded using a predefined framework adapted from established monitoring protocols [29], which was applied consistently to both outdoor and television advertisements.

##### Television advertising

Three nutritionist-researchers reviewed recorded broadcasts to identify commercial advertisements aired during program breaks. Product placements embedded within programs were excluded. The coders received standardized training, and pilot testing was conducted prior to the main coding phase. An initial subset of programming (4 h) was coded independently to assess inter-coder reliability. Agreement exceeded 90%, after which any discrepancies were discussed and the coding procedures were refined before independent coding of the full dataset.

For each identified advertisement, data were extracted using the INFORMAS protocol [29], a standardized tool developed to monitor food marketing practices globally. Information recorded included channel name, day of the week, program category, time slot and advertisement type (food or non-food). Food advertisements were defined as any commercial promoting food brands or products, including infant formula and food supplements.

Additional information was collected for food advertisements, including the product(s) promoted, brand name, and the presence of persuasive marketing techniques. The same predefined framework of persuasive marketing techniques (promotional characters, premium offers, brand benefit claims, health and/or nutrition-related claims) that was used for outdoor advertising was applied to ensure consistency across media.

##### Nutrient profiling

The WHO-NPM AFRO was used to classify advertised food and beverages, which defines 17 food categories and nutrient thresholds to determine whether products are ‘permitted’ or not for marketing to children [34]. When multiple products appeared in a single advertisement, each product was assessed separately.

Nutritional information was primarily obtained from a pre-existing database developed from a market survey conducted in Senegal [8]. Additional data for newly identified products were collected through targeted visits to retail outlets. Information extracted from product labels included energy content, total fat, saturated fat, total sugars, salt, sodium, fiber, protein content per 100 g/ml and the proportion of fruits, vegetables and legumes (FVL). Products lacking nutritional information and infant formulas were excluded from the nutritional analyses, as infant formulas fall outside the scope of the WHO-NPM AFRO.

Nutritional quality was assessed using the WHO-NPM AFRO and Nutri-Score systems. Under the WHO-NPM AFRO, products exceeding one or more category-specific nutrient thresholds were classified as ‘non-permitted’ for marketing to children. Nutri-Score assigns foods a score ranging from −15 (highest nutritional quality) to +40 (lowest nutritional quality), based on nutrient composition and FVL content. Products are then classified into one of the five categories, ranging from A (healthiest) to E (least healthy) [35].

### Data analysis

The outdoor and television advertising datasets were analyzed separately using descriptive statistics, including medians (interquartile ranges) or proportions, as appropriate. For outdoor advertising, the distribution of food advertisements according to nutritional classification was assessed across geographic areas (urban vs. peri-urban) and school characteristics (primary/secondary and public/private). For television advertising, the rate of food advertisements per hour, stratified by nutritional quality (permitted vs. non-permitted) was compared across days of the week (weekdays vs. weekends) and viewing periods (peak vs. non-peak hours). Chi-square tests were used to assess differences in the distribution of food advertising. A *p*-value of less than 0.05 was considered statistically significant. All quantitative analyses were performed using R (version 4.2.1) and RStudio.

Qualitative data were analyzed using thematic analysis, following the six-phase approach described by Braun and Clarke [36]. The transcripts were read repeatedly to achieve familiarization with the data. An initial coding framework, informed by the interview guide topics, was used to develop the early stages of the analysis process and was iteratively refined as new concepts emerged from the data. Coding was performed by two members of the research team. These codes were refined and organized into broader themes that captured recurring patterns and meanings across participants’ experiences.

## Ethical considerations

The study was conducted in accordance with the Declaration of Helsinki and received ethical approval from the National Ethics Committee for Health Research (CNERS) of the Ministry of Health (approval No. SEN2021/78). Prior to data collection, approval was obtained from school directors and informed consent was obtained from all adult participants. For children and adolescents, written consent from a parent or legal guardian consent, as well as assent from the participant, was obtained prior to data collection. All participants were informed about the study objectives, the voluntary nature of participation and their right to withdraw from the study at any time without consequence. To preserve participants’ confidentiality, the study sites are anonymized throughout the qualitative findings and referred to as sites 1, 2, 3 and 4.

## Results

### Extent of food advertising around schools

A total of 684 food and beverage advertisements were identified within a 250-m radius of the 40 selected schools, with a marked concentration in Dakar Region, which accounted for over two-thirds of all food advertisements (*p* < 0.001).

Food advertising was significantly more prevalent in urban compared to peri-urban municipalities, with higher densities observed around secondary, mixed and private schools (*p* < 0.001). The overall median density was 14.5 advertisements per school [IQR: 2.7–23.7], indicating substantial commercial presence in school environments. These patterns suggest unequal distribution of food marketing, with greater concentration in more urbanized settings (Table 1).

**Table 1.** Distribution and density of food and beverage advertisements around schools, Senegal.

Variable		Food advertisements n (%)	Median number of ads per school (IQR)	*p-value*
**Total**		684	14.5 [2.7–23.7]	–
**Region**				<0.001
Dakar		474 (69.3)	24.5 [18.0–40.5]	
Kaolack		40 (5.8)	3.0 [0.7–7.2]	
Saint-Louis		81 (11.8)	7.0 [1.7–14.2]	
Ziguinchor		89 (13.0)	4.0 [1.5–10.2]	
**Geographical area**				<0.001
Urban		437 (63.9)	19.0 [7.5–30.3]	
Peri-urban		247 (36.1)	13.0 [0.7–18.7]	
**School level**				<0.001
Elementary		187 (27.3)	12.0 [1.0–19.0]	
Secondary		313 (45.8)	13.0 [5.2–24.7]	
Mixed		184 (26.9)	23.0 [12.0–39.5]	
**School status**				<0.001
Public		301 (44.0)	10.0 [2.7–21.5]	
Private		383 (56.0)	18.0 [5.0–25.0]	

n indicate the number of advertisements; Value are presented as n(%) or median (IQR). *p*-values refer to differences across regions, area type, and school characteristics IQR: interquartile range.

### Nutrient profiling of food advertisements around schools

Of the food and beverages advertisements identified around schools, 107 were excluded from nutrient profiling analysis, including advertisements related to baby foods (*n* = 14), brand-only promotions (*n* = 45), and products for which no nutritional information was available (*n* = 48). The nutritional quality of advertised foods was consistently poor across settings, with most products classified as ‘non-permitted’ under the WHO-NPM AFRO. Clear spatial disparities emerged, with peri-urban areas showing a higher concentration of ‘non-permitted’ foods and a predominance of Nutri-score E products, pointing to a clustering of nutritionally poor food advertising in more disadvantaged environments. Variations were also observed across school categories, with primary school environments showing a higher proportion of advertisements promoting nutritionally poor foods and beverages. Differences by school status were limited, indicating that unhealthy food marketing is widespread across educational settings (Table 2).

**Table 2.** Classification of food and beverage advertisements around schools according to WHO-NPM AFRO criteria and Nutri-Score classification, by geographical area and school characteristics, Senegal.

Foods advertising	WHO-NPM AFRO n (%)			Nutri-Score classification n (%)						
	Permitted 118 (21.2)	Non-permitted 438 (78.8)	p-value	Nutri-Score A 41 (7.4)	Nutri-Score B 76 (13.7)	Nutri-Score C 24 (4.3)	Nutri-Score D 27 (4.8)	Nutri-Score D/E 11 (2.0)	Nutri-Score E 377 (67.8)	p-value
**Geographical area**			<0.001							p < 0.01
Urban (*n* = 352)	88 (25.0)	264 (75.0)		34 (9.7)	51 (14.5)	20 (5.7)	17 (4.8)	10 (2.8)	220 (62.5)	
Peri-urban (*n* = 204)	30 (14.7)	174 (85.3)		7 (3.4)	25 (12.3)	4 (2.0)	10 (4.9)	1 (0.5)	157 (77.0)	
**School level**			<0.001							p = 0.42
Elementary (*n* = 151)	18 (11.9)	133 (88.1)		3 (2.0)	18 (11.9)	7 (4.6)	7 (4.6)	7 (4.6)	109 (72.2)	
Secondary (*n* = 259)	67 (25.9)	192 (74.1)		24 (9.3)	38 (14.7)	14 (5.4)	12 (4.6)	4 (1.5)	167 (64.5)	
Mixed (*n* = 146)	33 (22.6)	113 (77.4)		14 (9.6)	20 (13.7)	3 (2.1)	8 (5.5)	0 (0.0)	101 (69.2)	
**School status**			<0.001							p = 0.15
Public (*n* = 246)	60 (24.4)	186 (75.6)		20 (8.1)	36 (14.6)	13 (5.3)	14 (5.7)	11 (4.5)	152 (61.8)	
Private (*n* = 310)	58 (18.7)	252 (81.3)		21 (6.8)	40 (12.9)	11 (3.5)	13 (4.2)	0 (0.0)	225 (72.6)	

Percentage are based on the total number of food and beverage advertisements identified around schools. Products with missing nutritional information were excluded from nutrient profiling analyses. *p*-values from chi-square tests assess whether the distribution of advertisements according to WHO-NPM AFRO and Nutri-Score classifications differs by geographical area, school level and school status. Values < 0.05 were considered statistically significant.

### Frequency of advertisements on television channels

A total of 576 h of television broadcasting were recorded across the four selected channels (144 h per channel). Overall, of the 1744 advertisements that were identified, food and beverage advertising accounted for over one-quarter (28.3%).

Advertising was heavily concentrated on private channels, which broadcast substantially higher volumes and rates of both total and food advertisements compared to the public channel (*p* < 0.001). Higher food advertising frequency was also observed during weekends relative to weekdays, although differences were modest (*p* < 0.05) (Table 3).

**Table 3.** Average rate of total and food and beverage advertisements per hour per channel, by television channel and day of the week, Senegal.

Channel	All advertisements n (%)	Mean rate (ads/hour/channel) [95% IC]	Food and beverage advertisements n (%)	Mean rate (ads/hour/channel) [95% IC]
Total	1 744	3.0 [2.8–3.2]	493 (28.3)	0.9 [0.8–0.9]
Public channel 1	190 (10.9)	1.3 [1.1–1.5]	29 (5.9)	0.2 [0.1–0.3]
Private channel 1	495 (28.4)	3.4 [3.1–3.8]	124 (25.1)	0.9 [0.7–1.0]
Private channel 2	670 (38.4)	4.6 [4.3–5.0]	201 (40.8)	1.4 [1.2–1.6]
Private channel 2	380 (21.8)	2.6 [2.4–2.9]	139 (28.2)	1.0 [0.8–1.1]
*p*-value	<0.001		<0.001	
Weekday	825 (47.3)	2.9 [2.7–3.1]	223 (45.2)	0.8 [0.7–0.9]
Weekend	919 (52.7)	3.2 [3.0–3.4]	270 (54.8)	0.9 [0.8–1.1]
*p*-value	0.024		0.034	

Values are presented as percentage or mean rate with 95% confidence intervals (CI). Rates are expressed as advertisements per hour per channel (ads/hour/channel); *p*-values indicate differences across television channels and between weekdays and weekend days.

### Most promoted foods categories around schools and on television channels according to WHO-NPM AFRO

Around schools, most advertisements promoted a single product and were predominantly promoted by packaged food and non-alcoholic beverages manufacturers, with limited contribution from retailers and quick-service restaurants. Brand-only promotions represented a small proportion of advertisements in both school and television settings. Across both settings, energy drinks were the most frequently promoted category, followed by milk and dairy-based drinks, tea and coffee while sauces, dips and dressings, and edible oils were prominent on television (Table 4).

**Table 4.** Distribution of food and beverage advertisements according to WHO-NPM AFRO categories, around schools and on television channels.

	On TV channels (*n* = 493 ads)		Around schools (*n* = 684 ads)	
Food categories	nc = 542	Frequency of advertising %	nc = 578	Frequency of advertising %
Beverages	341	62.9	376	65.0
a)Energy drink	224	41.3	228	39.4
b)Coffee, tea, herbal infusion	39	7.2	74	12.8
c)Milk and dairy based drinks	60	11.1	72	12.4
d)Juices	18	3.3	2	0.3
Flan, custard, yoghurt	2	0.4	45	7.8
Sauces, dips and dressings	91	16.8	40	6.9
Frozen yoghurt, ice cream and sorbets	–	–	25	4.3
Butter, oils and emulsions	82	15.1	24	4.1
Chocolate and sugar confectionery	5	0.9	24	4.1
Processed fruits, vegetables and legumes	6	1.1	16	2.8
Pasta, noodles, rice	7	0.9	9	1.5
Processed meats	–	–	7	1.2
Processed fish and seafood products	–	–	7	1.2
Cheese			2	0.3
Cakes, sweet biscuits and pastries	8	1.5	2	0.3
Breakfast cereals	–	–	1	0.2
Brand-only	25	5.1	45	6.6

n represents food and beverages advertisements; nc represents the number of products categories listed in food advertisements.

### Characteristics of food advertisements on television channels according to the nutrient profiling system

**Figure 1.**
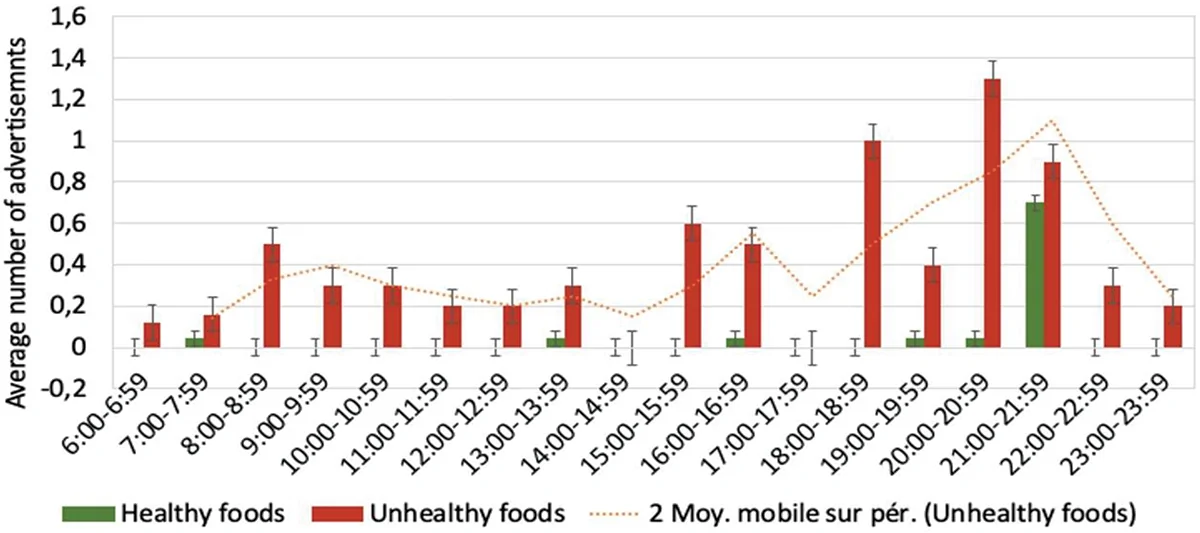
Average number of food advertisements per hour according to their nutritional quality on weekdays.

**Figure 2.**
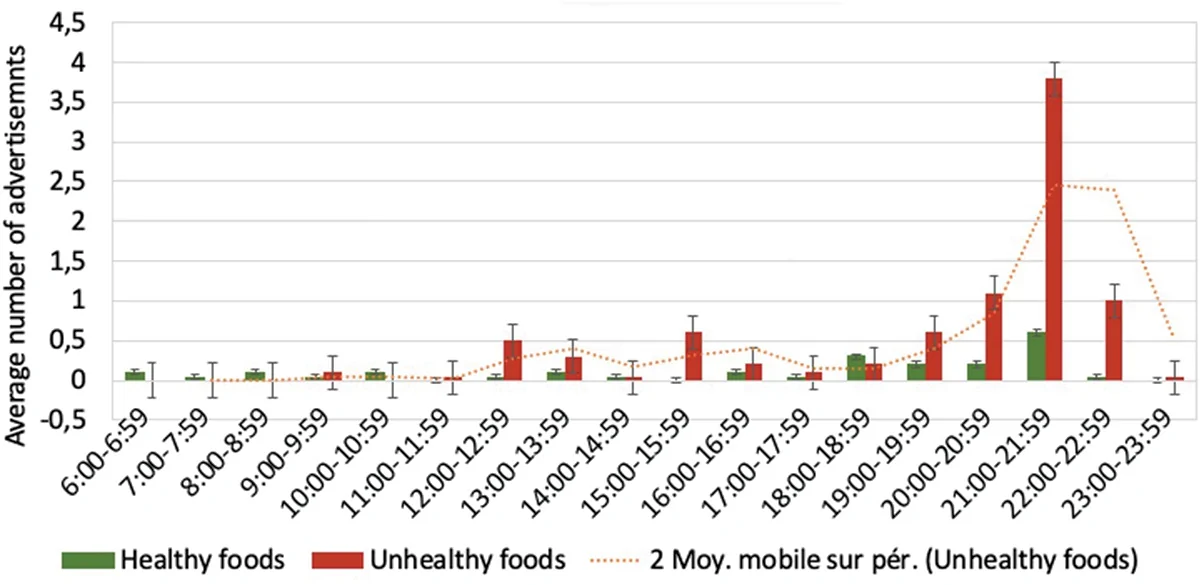
Average number of food advertisements per hour according to their nutritional quality on weekend day.

Overall, foods and beverage advertising on television was substantially higher during peak viewing time than during non-peak viewing periods (Table 5). Across both periods, ‘non-permitted’ foods accounted for most advertisements and consistently showed higher advertising rates than ‘permitted’ foods (Figures 1 and 2). Similarly, lower nutritional quality products (Nutri-Score D and E) dominated advertising, particularly during peak viewing time.

**Table 5.** Average rate of advertising according to peak viewing time (PVT) and non-peak viewing time (PVT).

	Food and beverage advertisements n (%)	Peak viewing time		Non-peak viewing time	
		n (%)	Ads/h	n (%)	Ads/h
**All**	493	271 (55.0)	1.4 [1.2–1.6]	163 (33.1)	0.4 [0.4–0.5]
**WHO-NPM AFRO (*n* = 434)**					
Permitted foods	68 (15.7)	41 (15.1)	0.2 [0.1–0.3]	27 (16.6)	0.1 [0.0–0.1]
Non-permitted foods	366 (84.3)	230 (84.9)	1.2 [1.0–1.4]	136 (83.4)	0.3 [0.3–0.4]
*p*-value	<0.001	<0.001		<0.001	
**Nutri-Score (*n* = 425)**					
Nutri-Score A	37 (8.7)	15 (5.5)	0.1 [0.0–0.1]	22 (13.5)	0.0 [0.0–0.1]
Nutri-Score B	10 (2.3)	6 (2.2)	0.0 [0.0–0.1]	4 (2.4)	0.0 [0.0–0.0]
Nutri-Score C	25 (5.9)	23 (8.5)	0.1 [0.1–0.2]	2 (1.2)	0.0 [0.0–0.0]
Nutri-Score D	66 (15.5)	22 (8.1)	0.1 [0.1–0.2]	44 (27.0)	0.1 [0.1–0.1]
Nutri-Score E	287 (67.5)	201 (74.2)	1.0 [0.9–1.2]	86 (52.8)	0.2 [0.2–0.3]
*p*-value	<0.001	<0.001		<0.001	

Ads/h: advertisement per hour; *p*-values refer to chi-square tests comparing distribution across viewing periods.

### Nutritional quality of foods promoted around schools and on national television channels

Nutritional composition information was unavailable for 73 products (21.4%), which were excluded from nutrient profiling analyses. Overall, 76.2% (*n* = 260) of products were assessed according to WHO-NPM AFRO criteria and 77.4% (*n* = 264) according to the Nutri-Score classification.

**Figure 3.**
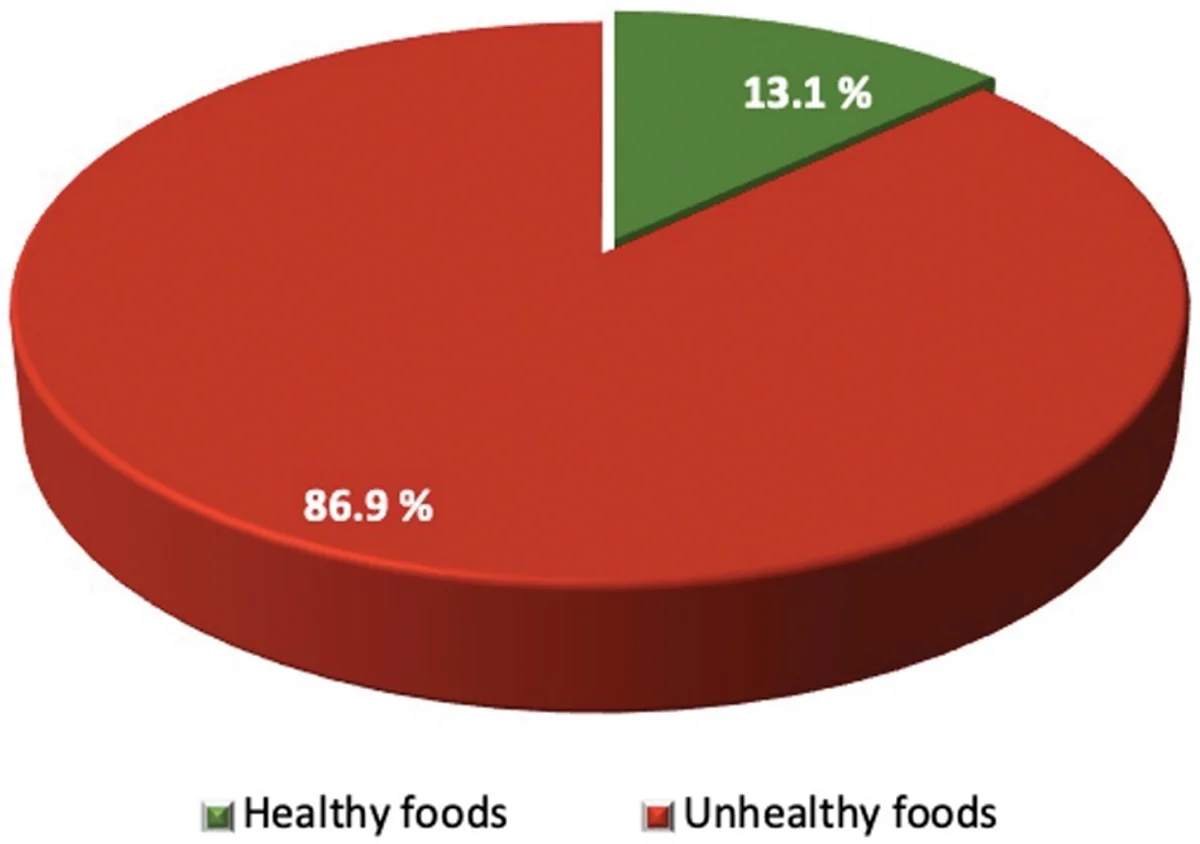
Nutritional quality of food promoted according to the WHO-NPM AFRO.

**Figure 4.**
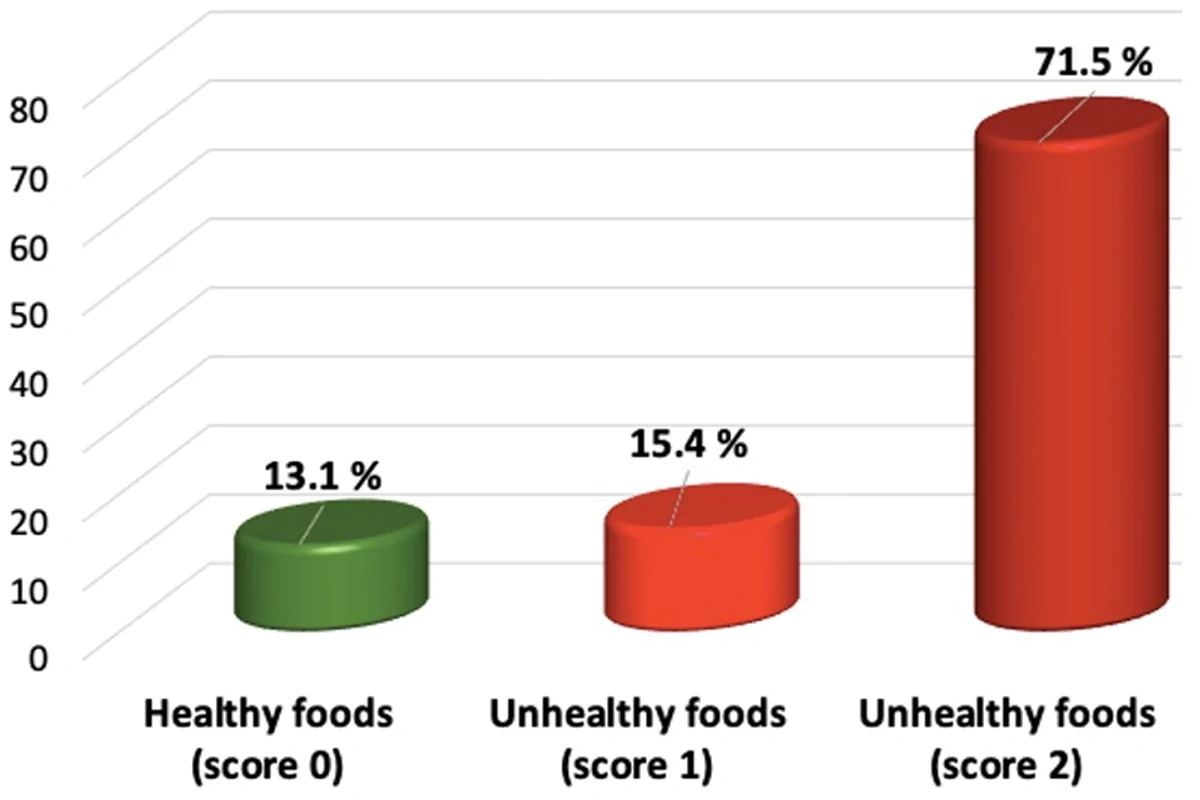
Compliance of promoted foods according to the WHO-NPM AFRO criteria.

**Figure 5.**
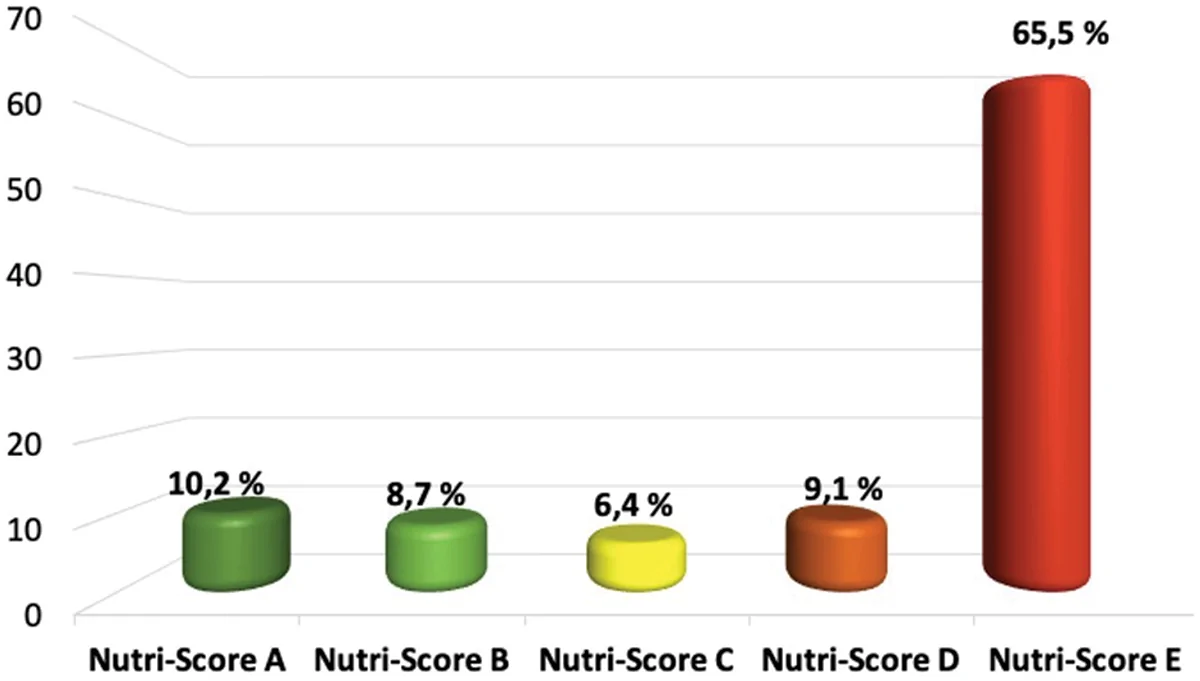
Nutritional quality of food promoted according to Nutri-Score classification system.

According to the WHO-NPM AFRO, most products were classified as ‘non-permitted’ (87%), with the majority exceeding thresholds for multiple nutrients of concern (Figures 3 and 4). Similar findings were observed using Nutri-Score, with approximately three-quarters of products classified in the lowest nutritional quality categories (D and E) (Figure 5).

### Persuasive marketing techniques

Persuasive marketing strategies were more frequently used in advertisements for ‘non- permitted’ foods compared with ‘permitted’ foods (Table S3, Table S4). The most commonly identified strategies included the use of cartoon characters or brand mascots, child-oriented characters, celebrity endorsements, and nutrient content claims.

## How children and parents experience and respond to unhealthy food marketing?

Four overarching themes were identified: (i) Pervasive and misleading advertising of unhealthy products (ii) Food advertising as a driver of food preferences and consumption practices, (iii) The persuasive power of marketing strategies, and (iv) Support for stronger regulation of unhealthy food advertising.

### Pervasive and misleading advertising of unhealthy products

Participants identified various sources of food advertising, including television (particularly around popular programs and news broadcasts), social media, online platforms, and outdoor billboards and posters located around schools. Exposure through digital and audiovisual media was frequently mentioned, with adolescents noting the pervasive nature of advertising across platforms. As one participant explained, *‘On social networks, every time we open a page, we see food adverts at the top of the list’* (FG4, site 1). Similarly, exposure to food and beverage advertising on television was described as being high, particularly during peak viewing times, such as early evening programs and new broadcasts.

Children demonstrated strong recall of advertised products and promotional messages. Across FGDs and interviews, participants consistently perceived that the majority of advertised products were unhealthy. Commonly cited items included sugar-sweetened beverages, chocolate spreads, dairy-based drinks, and culinary bouillons. These products were often described as excessively sweet or containing artificial additives and colourings. As one participant noted, *‘bouillons and powdered juices … contain colourings, they shouldn’t be advertised’* (FG2, site 1). Advertisements were also perceived as misleading, particularly in relation to health and nutrition claims. For example, one adolescent stated *‘sometimes, in adverts, they say that drinks contribute to growth … but it’s not true’* (FGD18, site 3), highlighting scepticism towards promotional messages.

Participants further emphasized that advertising content was predominantly targeted at children and focused on highly palatable products. As one participant described, *‘the foods advertised are generally sweet products, aimed at children’* (FGD20, site 3). Some children reported requesting advertised products from their parents’ following exposure on television, suggesting a direct influence on purchase demands and food preference.

### Food advertising as a driver of food preferences and consumption practices

Children and adolescents widely acknowledged that food advertising influences their food preferences and purchasing behaviours. Several children and adolescents reported purchasing products immediately after exposure to television advertisements, indicating a strong persuasive effect. As one participant stated, ‘*when I saw Dolima milk in the advert, I bought it’* (FG19, site 3), illustrating how advertising can directly translate into consumption behaviour.

In some cases, purchasing was driven by curiosity and a desire to verify advertising claims through personal experience. One adolescent explained, ‘*I wanted to know if what they were saying was true or not. But when I tasted the Laicran, I noticed that it was delicious and it also gave me energy, which is why I only drink Laicran’* (FGD16, site 3), suggesting that initial scepticism may be resolved through positive sensory experience, which in turn can reinforce brand preference and loyalty. Similarly, another participant reported purchasing a product after exposure to advertising, stating, *‘once I bought it just because I saw the advert’* (FGD13, site 3), highlighting the role of advertising in exploratory consumption.

Some participants reported disappointment when products failed to meet expectations created by advertising. Concerns related to exaggerated taste, quality and health claims were frequently mentioned. As one adolescent noted, *‘in juice ads, they say they are delicious, but when you buy them, you realize there is too much sugar and coloring’* (FGD8, site 1), highlighting a perceived mismatch between promotional claims and lived consumption experiences.

### The persuasive power of marketing strategies

Participants identified a range of persuasive marketing strategies used by food companies to attract children and adolescents. These included the use of music and catchy slogans, celebrities and sports endorsements, the presence of children in advertisements, and promotional incentives such as discounts and special offers.

Music and slogans were frequently mentioned as particularly memorable and emotionally engaging. For example, one child recalled the slogan *‘Bridel et la vie est belle’* [Bridel and life is beautiful] (FGD4, site 1). A parent also highlighted the intentional use of sound-based strategies, noting, *‘they put a sound so that when children hear this song, they want the product’* (IDI3, site 1). The use of children in advertisements was also perceived as a trust-building strategy. As one child explained, ‘they show children consuming the products, which give us confidence’ (FGD10, site 1), indicating that peer representation enhances perceived credibility and acceptability.

Celebrity and sports endorsements were widely recognized as influential, particularly among boys. Participants mentioned prominent athletes such as ‘Balla Gaye with his Crax crips ad’ and ‘Eumeu Sene with his 3X ad’ (FGD18, site 3). Similarly, international celebrity endorsement was seen as highly persuasive, with one adolescent stating, *‘I love the Pepsi advert because it’s Messi doing the advertising’* (FGD9, site 1), highlighting the aspirational role of global sports figures in shaping brand appeal.

Promotional offers also played a significant role in shaping consumption decisions. One adolescent noted, *‘I like KFC and when there’s a discount on food like KFC, I buy it’* (FGD20, site 3), illustrating the influence of price-based incentives on purchasing behaviour.

### Support for stronger regulation of unhealthy food advertising

Participants, particularly parents, advocated strong support for stricter regulation of food advertising, especially for products perceived as unhealthy. They called for tighter advertising controls, public awareness campaigns on the health risk associated with unhealthy diets, and mandatory health or nutrition warnings within advertisements to better inform the population, particularly children. As one parent emphasized, *‘the Government must ensure that good food is marketed and not unhealthy food’* (ID17, site 3), highlighting expectations for stronger state intervention in shaping the food environment.

Concerns about the health consequences of unhealthy diets were frequently expressed, with participants advocating for restriction on the promotion of products high in sugar, fat, and additives. As one parent stated, *‘we should avoid spreading food with sugar and colourings’* (ID4, site 1), another added, *‘you mustn’t advertise bouillons’* (IDI21, site 3). Similarly, adolescents suggested that certain products should not be promoted, noting”, *‘drinks containing too much sugar should not be advertised’* (FGD15, site 2).

Some participants emphasized the need for early-life awareness and behavioral change, linking advertising regulation to broader public health education. One adolescent explained, ‘*we have to stop drinking all these drinks … make young people aware from an early age, show them the consequences of an unhealthy diet*’ (FGD8, site 1), reflecting a preventive and education-oriented perspective. Others children highlighted the importance of restricting specific categories of foods, stating, *‘certain foods should not be advertised such as very sweet lollipops, Sama Dom crisps, King drinks … we must avoid advertising here in Senegal to save many children’* (FGD19, site 3), underscoring a perceived protective role of regulation.

Participants also expressed support for promoting healthier dietary behaviours and positive messaging. As one adolescent suggested, *‘avoid snacking between meals’* (FGD16, site 2), others proposed that advertising should prioritize healthy food options such as fruits, vegetables and organic products. The use of positive health messaging was also perceived as an effective communication strategy. This was illustrated by reference to preventive health campaigns: *‘I’ve seen dentists advertising on TF1 to encourage children to brush their teeth every day’* (FGD6, site 1), suggesting the potential for health-promoting advertising to serve as a model for counterbalancing the promotion of unhealthy foods.

## Discussion

This study provides the first comprehensive evidence in Senegal of the extent and nature of food advertising in children’s environments, combining a quantitative assessment across television and schools’ settings with qualitative insights into children’s and parents’ perceptions. By integrating these approaches, the study documents the scale of marketing but also elucidates the behavioral pathways through which advertising may influence children’s food preferences and consumption patterns.

The findings indicate a pervasive presence of food advertising promoting low-nutrient products in both physical and virtual environments. The predominance of ‘non-permitted’ foods under the WHO-NPM AFRO, alongside the high proportion of Nutri-score D and E products, indicates an obesogenic environment in children’s daily settings. These findings are consistent with evidence from other African countries [19–22,37] and global systematic reviews showing that children are disproportionately exposed to the promotion of nutrient-poor, energy dense products [38].

This study highlights a patterned distribution of advertising, with higher concentrations observed in urban settings and during peak television viewing times. This temporal and spatial clustering increases the likelihood of repeated exposure to marketing of unhealthy foods, which has been associated with increased brand recognition, food preferences, purchase requests and consumption of these foods [33,39]. These finding indicate a systematic temporal concentration of advertising for nutrient-poor foods during periods of higher audience exposure. This suggests that time-based regulatory strategies may be relevant to reduce prevalence of unhealthy food advertising. This is particularly relevant in Senegal, where existing advertising regulations remain largely general in scope and no specific restrictions currently address the marketing of HFSS foods and beverages to children.

Qualitative findings provide further insight into the food advertising context in Senegal, showing that children recall brands, experience cravings, and actively request advertised foods. Parents also acknowledge the influence of marketing on their purchasing decisions. These findings demonstrate how marketing exposure translates into behavioral responses, shaping food preferences and consumption practices [15,16].

The predominance of energy drinks across both settings is particularly concern, given the evidence linking their consumption to excess sugar intake and diet-related NCDs [40]. These patterns reflect broader shifts in food environments associated with the nutrition transition [41] and highlight the need for preventive policy responses targeting the marketing of such unhealthy foods.

The study highlights the persuasive power of marketing techniques. ‘Non-permitted’ foods were more likely to be promoted using celebrity endorsements, attractive imagery and catchy slogans. Children and adolescents demonstrated strong brand recall and described these techniques as influential in terms of their purchasing patterns. As children’s cognitive capacity to critically interpret persuasive intent is still developing [25], such strategies can facilitate the development of brand loyalty, cravings, and repeated purchase requests [16]. These mechanisms represent a key pathway through which food marketing contributes to the development of unhealthy dietary patterns overtime.

Our findings also highlight structural constraints that limit the effectiveness of individual-level awareness. Although parents expressed awareness of the health risks associated with unhealthy food and supported for regulatory measures, their purchasing decisions were influenced by pervasive marketing and their children’s purchase requests. This reinforces the need to move beyond education-based approaches towards structural interventions that modify food environments.

International evidence suggest that mandatory regulatory approaches are more effective than voluntary self-regulation in reducing children’s exposure to unhealthy food marketing and improving food environments [12,42]. Recent WHO normative guidance further emphasizes that restricting such marketing is a core component of comprehensive strategies to create healthier food environments for children. Meanwhile, UNICEF emphasizes the need for comprehensive restrictions on marketing covering all media channels, including television, outdoor advertising, and digital platforms, to effectively protect children from pervasive and persuasive marketing practices [6,25].

Our findings suggest an urgent need to strengthen the policy and regulatory framework governing food marketing to children in Senegal. Current evidence indicates that regulatory measures to improve food environments remain limited [43]. Important priorities for protecting children and adolescents from unhealthy food marketing include mandatory restrictions on the marketing of ‘non-permitted’ foods across physical and media environments, supported by effective monitoring and enforcement mechanisms.

## Strengths and limitations

This study has several strengths. It provides the first comprehensive assessment in Senegal of food advertising across both physical and media environments relevant to children, integrating outdoor advertising mapping around schools with systematic television monitoring. The use of a mixed-methods approach further strengthens the analysis by integrating quantitative measures of advertising prevalence with qualitative insights into children’s and parents’ perceptions. In addition, the application of standardized nutrient profiling models, including the WHO-NPM AFRO and the Nutri-Score classification, enhances the comparability of findings with international evidence and strengthens their policy relevance.

However, some limitations should be considered when interpreting the findings. First, the use of a 250-metre buffer around schools, while consistent with similar studies, may have resulted in overlapping zones in some cases. Although advertisements within these areas were recorded only once to avoid duplication, this approach may have led to an underestimation of advertising density at the individual school level.

Second, television advertising was recorded over a short three-month period and channels were not monitored simultaneously. While this approach allowed for a structured assessment across channels, it may not fully capture seasonal variation or temporal fluctuations in advertising trends throughout the full year.

## Conclusion

This study demonstrates that Senegalese children and adolescents are widely exposed to advertising for unhealthy food across physical and virtual environments. Most of the products promoted are classified as non-permitted under the WHO NPM-AFRO. Converging quantitative and qualitative findings reveal that the marketing of unhealthy food is pervasive and persuasive, influencing children’s food preferences and purchasing behaviors.

These findings emphasize the urgent need to strengthen Senegal’s regulatory framework governing food marketing to children. Priority actions include implementing mandatory restrictions on the marketing of non-permitted foods on television and in outdoor environments surrounding schools, as well as on digital media. These measures should be supported by the development of a context-specific nutrient profiling model to inform regulatory decisions, the implementation of front-of-pack nutrition labelling to strengthen existing nutrition labelling, and effective monitoring and enforcement mechanisms. As part of a wider package of policies to create a healthier food environment, including fiscal and other evidence-based interventions, these actions could contribute to healthier dietary choices and the prevention of diet-related non-communicable diseases among children and adolescents in Senegal.

## Supplementary Material

SUPPLEMENTARY_MATERIAL_GHA_clean.docx

## Data Availability

Data is available upon reasonable request from the corresponding author.
