# Supplementary material for: Children’s exposure to unhealthy food and beverage advertising in Senegal: a cross-sectional mixed-methods analysis of advertising trends, marketing strategies and perceptions
Source: Glob Health Action. 2026 Sep 4;19(1):2723719. doi: 10.1080/16549716.2026.2723719 (PMC13552323; doi:10.1080/16549716.2026.2723719)
Supplement: Supplementary file 1 [file ZGHA_A_2723719_SM3557.docx]

**Children’s exposure to unhealthy food and beverage advertising in Senegal: a cross-sectional mixed-methods analysis of advertising trends, marketing strategies and perceptions**

**Appendix – Supplementary Material**

**Figure S1: Television recording schedule across the 12-week monitoring period (May-July 2023)**

*Note: Ch. PB = Public television channel; Ch. PR = Private television channel; The four selected television channels (one public and three private) were recorded following a structured rotational schedule over eight non-consecutive days per channel. Television broadcasts were recorded between 6:00 am and 00:00 (midnight) on four weekdays (Monday, Tuesday, Thursday, Friday) and four weekend days (2 Saturday and 2 Sunday) over a 12-week period. The rotation ensured balanced coverage across different days of the week while minimizing duplication of programming content.*

**Table S1: Distribution of focus groups discussions and participants across regions**

|  | **Dakar** | **Kaolack** | **Saint-Louis** | **Ziguinchor** | **Total** |
| --- | --- | --- | --- | --- | --- |
| **Number of FGDs conducted** | 11 | 6 | 4 | 3 | 24 |
| Public schools | 6 | 3 | 2 | 2 | 13 |
| Private schools | 5 | 3 | 2 | 1 | 11 |
| **Participants in FGDs (n=209)** | 90 | 55 | 37 | 27 | 209 |
| Boys (7-12 years) | 26 | 9 | 9 | 9 | 53 |
| Girls (7-12 years) | 17 | - | 10 | - | 27 |
| Adolescent boys (13-17 years) | 14 | 10 | 9 | 18 | 51 |
| Adolescent girls (13-17 years) | 33 | 36 | 9 | - | 78 |
| **Number of in-depth interviews conducted** | 11 | 4 | 7 | 4 | 26 |
| Teachers and school directors | 7 | 3 | 3 | 2 | 15 |
| Parents | 1 | 1 | 3 | 1 | 6 |
| Food vendors | 3 | - | 1 | 1 | 5 |

*Note: FGD = focus group discussion. Participants were recruited from a sub-sample of the selected schools across the four study regions. Participants numbers represent individuals who took part in focus group discussions or in-depth interviews. Children were defined as participants aged 7-12 years and adolescents as those aged 13-17 years. In depth interviews included teachers, school directors, parents or caregivers and food vendors. A dash (-) indicates that no participants were included in the corresponding category*.

**Table S2: Description of the foods categories based on the WHO Nutrient Profile Model for the African Region (WHO-NPM AFRO)**

| **Foods categories** | **Description of the foods** |
| --- | --- |
| **Bread and bakery products** | |
| a) Cakes, sweet biscuits and pastries | Sweet bakery products such as cakes, cookies, doughnuts, muffins, sweet pancake, croissants, etc. |
| b) Bread and crisp bread | Bread and rolls, wraps, tortillas, bread with raisins, bread with cereals, mixes for making bread, pizza, etc. |
| **Savoury snack foods** | |
| a) Savoury snacks Potato, cereal, starch-based | Popcorn, chips, crisps, other snacks made from rice, maize, wheat, potato, plantain, etc. |
| b) Processed nuts, edible seeds | Nuts, and mixed nuts including with fruit content, edible seeds |
| **Beverages** | |
| a) Juices | 100% fruit and vegetable juices prepared from fresh or reconstituted from concentrate, smoothies, etc. |
| b) Milk and dairy based drinks | Milk and sweetened milks, reconstituted powdered milk, flavoured dairy products (chocolate milk, strawberry milk, cocoa), condensed milk, milk shakes |
| c) Energy drink | Electrolyte drinks, carbonated and non-carbonated water-based flavoured drinks, powdered juices, flavoured waters, reconstituted chocolate or malted powdered drnks, syrups, sugar cane juices, etc. |
| d) Coffee, tea, herbal infusion | Coffee including instant and premixed coffee, tea including instant and premixed tea, herbal infusion to be prepared or in ready-to-drink form |
| e) Cereal, legumes, grain beverages | Cereal, grain and tree nut-based beverages produced from the extracts of cereals, pulses and tree nuts (e.g. rice, almond, soya, oat-based beverages) |
| Breakfast cereals | Broken or flaked grains of rice and other cereals, wheat-based or maize-based breakfast cereals, oat meat, mueslis, etc. |
| Chocolate and sugar confectionery | Chocolate bars, chocolate spread, muesli bars and granola, chewing gum, soft jellied candies, sweet candies, etc. |
| Cheese | Processed cheese, cheeses analogues (parmesan, cheddar, edam, mozzarella, ricotta), cream cheeses, spreadable cheeses, cottage cheese, etc. |
| Frozen yoghurt, ice cream and sorbets | Edibles ices, ice cream, ice milk, frozen yoghurt, ice lollies and sorbets |
| Flan, custard, flavored yoghurt | Dairy based products that have been curdled by fermentation, acid, ect. and flavoured with sugar and other ingredients (e.g. flavoured cream type yoghurt, jellied milk, chocolate mousse, puddings), flan, custard. |
| Ready-made and convenience foods | Composite foods: mixtures of multiples components (e.g. meat, sauce, grain, cheese, vegetables). These include foods that require minimal preparation or the ready-to-serve meal from restaurants (e.g. pizzas, lasagna, soups, tinned spaghetti, ready-made sandwiches, etc.) |
| Butter, oils and emulsions | Vegetable oils and fats, butter, margarine and similar products (e.g. cooking oils from plant, nuts spread: peanut butter, etc.) |
| Pasta, noodles, rice | Fresh, precooked or dried noodles and pasta, rice paper, rice noodles, vermicelli made from wheat, tapioca, brick paper, etc. |
| Processed meat | Smoked ham, salted dried meat, salami, sausage, bacon, corned beef, smoked duck, canned meats, chicken nuggets, beef or chicken patty, liver pate, etc. |
| Processed fish and seafood products | Salted fish and sea food, brined fish, fermented fish and sea food, sardine or mackerel, dried shrimp. |
| Processed fruits, vegetables and legumes | Fruits and vegetables in vinegar, oil or brine, dried, coconut, coconut cream, marmalade, jams, canned fruits, vegetables and legumes, dried mushrooms, preserved or pickled fruits and vegetables, fermented legumes, etc. |
| Sauces, dips and dressing | Emulsified, non-emulsified mixes as concentrated, clear sauces, soybean-based seasoning and condiments (e.g. mayonnaise, salad dressing, onion dips, tomato ketchup, cheese sauce, bouillon cubes, seasoning powder, fish sauce, sweet chili sauce, barbeque sauce, etc. |

**Table S3:** Persuasive marketing strategies used on foods advertising around schools

|  | **Permitted foods**  **(n=17)** | **Non-permitted foods**  **(n=96)** | **p-value** |
| --- | --- | --- | --- |
| **Promotional characters** | 4 (23.5) | 53 (55.2) | 0.016 |
| Cartoon | 0 (0.0) | 8 (15.1) |  |
| Company-owned character | 1 (25.0) | 19 (35.8) |  |
| Celebrity | 0 (0.0) | 8 (15.1) |  |
| ‘For Kids’ | 1 (25.0) | 9 (17.0) |  |
| Awards won | 0 (0.0) | 11 (20.7) |  |
| **Premium offers** | 1 (5.9) | 7 (7.3) | 0.836 |
| **Brand benefit claims** |  |  |  |
| Sensory based | 15 (88.2) | 90 (93.7) |  |
| Emotive claims | 3 (17.6) | 28 (29.1) |  |
| Suggested use | 5 (29.4) | 23 (23.9) |  |
| Price | 5 (29.4) | 29 (30.2) |  |
| **Claims** | 4 (23.5) | 22 (22.9) | 0.957 |
| Nutrition claims | 1 (25.0) | 16 (72.72) |  |
| Health claims | 2 (50.0) | 11 (50.0) |  |

*Note: Values are presented as n (%). Percentages were calculated using the total number of permitted (n=17) and non-permitted (n=96) food advertisements, respectively. Advertisements could contain more than one persuasive marketing technique. Promotional characters included cartoons, company-owned characters, celebrities, “for kids” appeals. Claims included nutrition and health-related claims. P-values were derived from chi-square or Fisher’s exact tests with statistical significance at p < 0.05*

**Table S4:** Persuasive marketing strategies used on foods advertising on TV channels

|  | **Permitted food**  **(n=11)** | **Non-permitted foods**  **(n=39)** | **p-value** |
| --- | --- | --- | --- |
| **Promotional characters** | 11 (100.0) | 36 (92.3) | 0.01 |
| Cartoon | 0 (0.0) | 1 (2.7) |  |
| Company-owned character | 1 (9.1) | 2 (5.5) |  |
| Celebrity | 6 (54.5) | 29 (80.5) |  |
| ‘For Kids | 1 (9.1) | 21 (58.3) |  |
| Awards won | 0 (0.0) | 4 (11.1) |  |
| **Premium offers** | 0 (0.0) | 0 (0.0) | **-** |
| **Brand benefit claims** |  |  |  |
| Sensory based | 10 (90.9) | 36 (92.3) |  |
| Emotive claims | 10 (90.9) | 28 (71.8) |  |
| Suggested use | 6 (54.5) | 25 (64.1) |  |
| Price | 0 (0.0) | 3 (7.7) |  |
| **Claims** | 4 (36.4) | 10 (25.6) |  |
| Nutrition claims | 2 (50.0) | 8 (80.0) | 0.837 |
| Health claims | 2 (50.0) | 5 (50.0) |  |
